# Physiological and Biochemical Responses of Rainbow Trout (*Oncorhynchus mykiss*) of Different Body Sizes Under Varying Temperature and Salinity Conditions: An L_9_ Orthogonal Design

**DOI:** 10.3390/biology15161383

**Published:** 2026-08-13

**Authors:** Jiahe Guo, Yuanhao Ren, Changjian Li, Hanfeng Zheng, Zhongquan Jiang, Fengyuan Shen, Bo Qin, Na Ying, Siping Li, Tingting Lin, Yanming Sui, Lei Li

**Affiliations:** 1 National Engineering Research Center for Marine Aquaculture, Zhejiang Ocean University, Zhoushan 316022, China; gjh20021128@163.com; 2 Key Laboratory of East China Sea Fishery Resources Exploitation, Ministry of Agriculture and Rural Affairs, China, East China Sea Fisheries Research Institute, Chinese Academy of Fishery Sciences, Shanghai 200090, China; zhenghf@ecsf.ac.cn (H.Z.); zhongquanj@sjtu.edu.cn (Z.J.); dhsqb1026@126.com (B.Q.); xyz6622@126.com (N.Y.); lisiping@ecsf.ac.cn (S.L.); lintt@ecsf.ac.cn (T.L.); 3 Jiangsu (Marine) Salmon and Trout Science and Technology Innovation Center, Yellow Sea Laboratory for Blue Seed Industry, Yancheng 224000, China; suiyanming@ycit.edu.cn; 4 Lianyungang Ocean and Fishery Development Promotion Center, Lianyungang 222000, China; xyz66221@126.com; 5 Jiangsu Key Laboratory for Exploration and Utilization of Marine Wetland Biological Resources, Yancheng Institute of Technology, Yancheng 224051, China

**Keywords:** rainbow trout, salinity acclimation, body size, water temperature, oxidative status, phosphatase activity, nitrogen metabolism, orthogonal design

## Abstract

This study evaluated the endpoint biochemical responses and associated physiological status of rainbow trout (*Oncorhynchus mykiss*) of different body sizes during salinity acclimation under varying temperature and salinity conditions. An L_9_ (3^4^) orthogonal design was applied using three nominal body-size levels (100, 250, and 500 g), three temperatures (10, 15, and 20 °C), and three salinities (10, 20, and 30‰) over 60 days. Hepatic superoxide dismutase (SOD), alkaline phosphatase (ALP), and acid phosphatase (ACP) activities and plasma malondialdehyde (MDA), urea nitrogen (BUN), ammonia, alanine aminotransferase (ALT), and aspartate aminotransferase (AST) were measured. For inferential analysis, the three fish sampled from each tank were averaged to obtain one tank-level value, yielding 18 tank-level observations. Tank-level main-effects analysis showed that body size was significantly associated with ALP, ACP, MDA, BUN, ammonia, ALT, and AST. Temperature was significantly associated with SOD, ACP, MDA, BUN, and AST, whereas salinity was significantly associated with MDA, ALT, and AST. MDA and AST were the only endpoints associated with all three factors. The biochemical responses were pathway-specific rather than uniformly directional across treatment combinations. Because interactions could not be independently estimated in the L_9_ design, the identified main effects should be interpreted as orthogonal screening associations. Overall, body-size class was associated with the broadest range of endpoints and should be incorporated into future experimental assessments of rainbow trout within the tested salinity range of 10–30‰.

## Simple Summary

Rainbow trout farming is gradually expanding from freshwater systems to brackish and marine environments, but fish of different sizes may respond differently to changes in water temperature and salinity. In this study, rainbow trout assigned to three nominal body-size levels were maintained under nine temperature–salinity combinations for 60 days. At the end of the experiment, liver tissue and plasma samples were analyzed to assess antioxidant defense, phosphatase-related functions, amino acid metabolism, and nitrogen waste processing. The measured indicators did not change in a uniform direction among treatments. Tank-level analysis showed that body size was associated with a broader range of liver and plasma measurements than temperature or salinity. Temperature and salinity showed more selective associations, particularly with indicators related to lipid oxidation and amino acid metabolism. These findings indicate that body size should be incorporated into experimental designs and physiological assessments of rainbow trout under brackish and saline culture conditions, although further experiments are required to clarify interactions and identify suitable culture conditions.

## 1. Introduction

Rainbow trout (*Oncorhynchus mykiss*) is an economically important cold-water aquaculture species that is highly responsive to changes in water temperature and culture conditions [1]. With the continued expansion of cold-water aquaculture and increasing demand for suitable farming space, the transfer of rainbow trout from freshwater systems to brackish and marine environments has received growing attention. Cold-water marine areas, including parts of the Yellow Sea, have also been identified as potentially suitable locations for offshore salmonid aquaculture [2]. However, transfer from freshwater to water of elevated salinity alters the osmotic gradient between the fish and its surrounding environment, thereby disturbing water and ion homeostasis and inducing adjustments in tissue metabolism. The capacity of rainbow trout to acclimate to salinity is therefore influenced not only by external salinity but also by water temperature and the physiological characteristics associated with body size.

Exposure to increased salinity requires a substantial reorganization of osmoregulatory and energy-related processes. Following transfer to saline water, fish regulate body-fluid osmolality and ionic composition through coordinated responses involving the gills, kidneys, and endocrine system. These processes increase energetic demands and may subsequently affect antioxidant defense and intermediary metabolism [3,4]. Morgan and Iwama reported that salinity altered metabolic rate and ion regulation in juvenile rainbow trout and steelhead trout, the latter representing the anadromous life-history form of *O. mykiss* rather than a separate species [3]. Different salinity-acclimation protocols have also been shown to influence osmoregulatory performance and energy allocation in rainbow trout [4]. During seawater acclimation, the phospholipid fatty acid composition of the gills changes according to water temperature and acclimation stage, and corresponding adjustments also occur in hepatic and intestinal membrane lipids [5,6]. Salinity acclimation may further influence smolt development and subsequent seawater performance, cardiovascular regulation, and the somatotropic axis of rainbow trout [7,8,9].

Water temperature is another major environmental factor governing fish physiology. It regulates oxygen consumption, feeding, digestion, nutrient utilization, and overall energy expenditure [10]. As water temperature increases, the oxygen demand and metabolic load of rainbow trout generally increase accordingly [11]. Metabolomic and biochemical evidence from the liver also indicates that thermal stress can alter antioxidant status and metabolic processes in this species [12]. Under salinity-acclimation conditions, temperature additionally affects the phospholipid fatty acid composition of the gills, liver, and intestine [5,6]. Consequently, exposure to the same salinity may not elicit an equivalent physiological response under different thermal conditions, because temperature can also influence the rate at which osmotic and ionic adjustments occur.

Body-size class may further be associated with differences in the physiological responses of salmonids to saline environments. Fish of different sizes differ in organ development, energy reserves, metabolic allocation, and ion-regulatory capacity. Larger steelhead trout parr have been reported to maintain comparatively stable osmoregulatory status following seawater transfer [13]. In Atlantic salmon and rainbow trout, the development of seawater adaptability is also influenced by body size, temperature, and photoperiod [14,15]. Body-size classes may therefore represent distinct physiological states rather than simply differences in body mass. Examining body size together with temperature and salinity can provide a more comprehensive assessment of the relative influence of each factor on biochemical responses during salinity acclimation. The three nominal body-size levels were selected to represent clearly separated size classes across a broad body-mass range, thereby enabling an initial evaluation of associations between body-size class and endpoint physiological and biochemical responses across different temperature–salinity combinations.

Environmental changes may alter both antioxidant defense and innate immune-related enzyme activities in fish. Superoxide dismutase (SOD) is a major component of the antioxidant defense system and catalyzes the removal of superoxide radicals, whereas malondialdehyde (MDA), a product of lipid peroxidation, is commonly used as an indicator of oxidative status [16,17,18]. Alkaline phosphatase (ALP) and acid phosphatase (ACP) are involved in phosphate-group hydrolysis, lysosomal digestion, and nonspecific immune responses [19,20]. Alanine aminotransferase (ALT) and aspartate aminotransferase (AST) participate in amino acid transamination and are associated with tissue and energy metabolism. Their activities in blood may therefore provide supplementary information on the biochemical status of fish [21,22]. Because these biomarkers represent different physiological processes, their responses are not necessarily coordinated, and changes in a single biomarker should not be interpreted independently as evidence of tissue injury or overall environmental suitability.

Nitrogen metabolism is closely associated with these physiological processes. Most teleost fish, including rainbow trout, are primarily ammonotelic. Ammonia generated through protein and amino acid catabolism is transported through the circulation and excreted mainly across the gills. Blood ammonia concentration is therefore determined by the balance among ammonia production, tissue transport, and branchial excretion, all of which may be affected by salinity and acid–base regulation. Although urea accounts for a relatively small proportion of nitrogenous waste in most teleosts, rainbow trout possess functional pathways for urea production and excretion. Salinity and environmental ammonia concentrations can alter the transbranchial ammonia gradient, ammonia excretion, and urea nitrogen metabolism in rainbow trout [23,24,25,26]. Blood ammonia and blood urea nitrogen may consequently provide complementary information on nitrogen metabolism alongside aminotransferase activities. However, these variables should not be interpreted according to the clinical criteria commonly applied to mammals.

Previous studies on salinity acclimation in rainbow trout have mainly focused on osmoregulation, ion transport, membrane composition, thermal stress, or production performance, and most have examined these factors separately or in two-factor settings. Consequently, it remains unclear whether body-size class is associated with different relative endpoint biochemical responses when body size, temperature, and salinity are evaluated within a single screening framework. We hypothesized that body size, temperature, and salinity would exert different relative influences on oxidative status, phosphatase activity, transamination, and nitrogen metabolism. Accordingly, the present study employed an L_9_ (3^4^) orthogonal array comprising three nominal body-size levels (100, 250, and 500 g), three water temperatures (10, 15, and 20 °C), and three salinities (10, 20, and 30‰). At the end of the 60-day trial, hepatic SOD, ALP, and ACP activities and plasma MDA, urea nitrogen (BUN), ammonia, ALT, and AST were measured. The objectives were to characterize endpoint physiological and biochemical patterns across the nine treatment combinations and to compare the relative main effects of body size, temperature, and salinity. By evaluating biomarkers from several physiological pathways, the study also examined whether the responses were coordinated or pathway-specific. The orthogonal design was used as a screening approach and was not intended to estimate interactions among the three factors.

## 2. Materials and Methods

### 2.1. Experimental Fish and Acclimation

Rainbow trout (*Oncorhynchus mykiss*) were obtained from a commercial hatchery in Weifang, Shandong Province, China. Upon arrival, fish from the different body-size classes were maintained separately in indoor freshwater flow-through tanks and acclimated for 15 days. During acclimation, the water temperature was maintained at 15 ± 1 °C under a 12 h light:12 h dark photoperiod. Dissolved oxygen ranged from 7.8 to 10.0 mg/L, and pH was maintained between 7.1 and 7.5.

Fish were fed a commercial formulated diet containing at least 45% crude protein (Shandong Hanye Biotechnology Co., Ltd., Binzhou, China) twice daily at 09:00 and 17:00. The same feed formulation and manufacturer were used for all body-size groups, whereas pellet diameter was adjusted according to fish size. Fish in the small-size group were provided with 3 mm pellets, while those in the medium- and large-size groups were provided with 5 mm pellets. The daily ration was approximately 2% of body weight. Fish were fasted for 24 h before the start of the experiment. Use of a common feed formulation minimized dietary-composition differences among body-size groups, whereas pellet diameter was adjusted to accommodate differences in ingestion capacity.

Based on the orthogonal experimental design, fish were assigned to three predefined body-size levels of 100, 250, and 500 g. Before the experiment, 30 fish were randomly sampled from each body-size level and individually weighed. The actual initial mean body weights were 53.87 ± 11.60, 187.87 ± 36.41, and 549.23 ± 65.27 g for the 100, 250, and 500 g levels, respectively. Stocking numbers were adjusted according to the measured body weights to maintain a comparable initial biomass among tanks. Accordingly, 140, 40, and 14 fish were stocked per tank for the 100, 250, and 500 g body-size levels, corresponding to estimated initial biomasses of approximately 7.54, 7.51, and 7.69 kg/tank, respectively. Hereafter, the 100, 250, and 500 g designations refer to the nominal body-size factor levels rather than to the measured initial mean body weights.

### 2.2. Orthogonal Experimental Design and Husbandry Conditions

A three-factor, three-level L_9_ (3^4^) orthogonal array was used to examine the physiological and biochemical responses associated with body size, water temperature, and salinity. The standard L_9_ (3^4^) orthogonal array contains four three-level columns. In the present study, three columns were assigned to body size, water temperature, and salinity, respectively, whereas the fourth column was left unassigned. No fourth biological factor was included or interpreted. The predefined body-size levels were 100, 250, and 500 g; the temperature levels were 10, 15, and 20 °C; and the salinity levels were 10‰, 20‰, and 30‰. The three factors were arranged into nine treatment combinations. Each treatment combination was implemented in two separate 600 L tanks operated as unidirectional flow-through systems. The tank was considered the experimental unit for the environmental treatments. The treatment combinations are presented in Table 1.

Water temperature was adjusted to the designated level at a rate of 1 °C/day. After the target temperature had remained stable for 24 h, salinity was increased at a rate of 2‰/day until the designated level was reached. The 60-day experimental period commenced after both water temperature and salinity had reached their respective target levels.

Throughout the trial, fish were fed the same commercial feed formulation used during the acclimation period twice daily at 09:00 and 17:00, with pellet diameter adjusted according to body size as described in Section 2.1. The daily ration was approximately 2% of body weight and was adjusted according to fish biomass and feeding condition. Feeding behavior and general activity were monitored daily. Daily feed provision was recorded, but uneaten feed was not quantitatively recovered; therefore, actual feed intake and feed-conversion indices could not be calculated. Body mass was assessed at the beginning and end of the trial as part of routine husbandry, but growth performance was outside the scope of the present biochemical analysis. Mortality occurred during the experimental period, and dead fish were recorded and removed promptly. Treatment-level mortality percentages are provided in Supplementary Table S2 as contextual husbandry information and were not included in the inferential statistical analysis of the biochemical endpoints.

Water temperature and salinity were recorded during daily husbandry, whereas dissolved oxygen and pH were monitored routinely throughout the experimental period. Water temperature remained within 1.0 °C of the designated level in each treatment, and salinity remained within 1.0‰ of the target value. Dissolved oxygen remained above 8.0 mg/L, while pH remained approximately 7.4. Ammonia nitrogen and nitrite nitrogen were not detected during routine monitoring. The actual 60-day mean water temperature and salinity for each treatment combination are provided in Supplementary Table S3.

### 2.3. Sample Collection

At the end of the 60-day trial, fish were fasted for 24 h. Three fish were randomly sampled from each of the two tanks used for each treatment. These fish were treated as within-tank subsamples, yielding three subsamples per tank and six sampled fish per treatment. Fish were euthanized by immersion in an overdose of tricaine methanesulfonate (MS-222; Yufubao, Nanchang, China; 200 mg/L). Sample collection was initiated after the cessation of spontaneous movement and opercular activity. Blood was then collected from the caudal vein.

Immediately after collection, the blood was gently mixed with a commercial ACD anticoagulant solution (Sangon Biotech (Shanghai) Co., Ltd., Shanghai, China) at a 1:1 volume ratio. The anticoagulant contained 0.45 mol/L sodium chloride, 0.10 mol/L glucose, 0.03 mol/L trisodium citrate, 0.026 mol/L citric acid, and 0.01 mol/L disodium ethylenediaminetetraacetate. The anticoagulated blood samples were maintained under chilled conditions and transported to the analytical laboratory. Before biochemical analysis, plasma was separated from the anticoagulated blood samples by centrifugation.

Following blood collection, the liver was excised immediately. Liver samples were rinsed with ice-cold phosphate-buffered saline, blotted dry with filter paper, transferred to centrifuge tubes, immediately frozen in liquid nitrogen, and subsequently stored at −80 °C until analysis.

### 2.4. Biochemical Analyses

Biochemical analyses were conducted by Qingdao Standard Testing Co., Ltd. (Qingdao, China). A total of 54 liver samples and 54 anticoagulated blood samples were submitted for analysis, comprising six individual fish samples from each of the nine treatment groups. Individual analytical results for all samples were provided in test report No. KY-20250908-003N. Plasma separated from the anticoagulated blood samples was used for all blood biochemical assays. The final analytical values and measurement units reported by the testing laboratory were used for subsequent data processing and statistical analysis.

Liver samples were analyzed for superoxide dismutase (SOD), alkaline phosphatase (ALP), and acid phosphatase (ACP) activities. Plasma samples were analyzed for malondialdehyde (MDA), urea nitrogen (BUN), ammonia, alanine aminotransferase (ALT), and aspartate aminotransferase (AST). The final analytical values and measurement units reported by the testing laboratory were used for subsequent data processing and statistical analysis.

All variables were measured using commercial microplate assay kits (Jiangsu Aidisheng Biotechnology Co., Ltd., Yancheng, China) according to the manufacturer’s instructions. Liver tissue was homogenized in the corresponding extraction buffer and centrifuged, and the resulting supernatants were used for analysis. SOD activity was determined using the WST-8 method at 450 nm (catalog no. ADS-W-KY011). ALP, designated as AKP in the kit documentation, and ACP activities were determined using p-nitrophenyl phosphate as the substrate at 405 nm (catalog nos. ADS-W-ZM003-96 and ADS-W-ZM002-96, respectively). Plasma MDA was measured using the thiobarbituric acid method based on the absorbance difference between 532 and 600 nm (catalog no. ADS-W-YH002). Plasma urea nitrogen was determined using a urease-based colorimetric method at 625 nm (catalog no. ADS-W-N013-96), whereas plasma ammonia was determined using the indophenol-blue method at 630 nm (catalog no. ADS-W-N021). Plasma ALT and AST activities were measured using 2,4-dinitrophenylhydrazine-based colorimetric methods at 520 nm (catalog nos. ADS-W-AJS001-96 and ADS-W-AJS002-96, respectively).

SOD activity was expressed as U/g fresh tissue, whereas ALP and ACP activities were expressed as μmol/min/g fresh tissue. Plasma MDA and urea nitrogen concentrations were expressed as nmol/mL and mg/dL, respectively; plasma ammonia concentration was expressed as μmol/L; and plasma ALT and AST activities were expressed as nmol/min/mL.

### 2.5. Statistical Analysis

The tank was considered the experimental unit. For each biochemical variable, the three fish sampled from the same tank were treated as subsamples and averaged to obtain one tank-level value. Thus, two tank-level observations were available for each treatment combination, resulting in 18 observations for inferential analysis. Treatment data are presented as the mean ± standard deviation of the two tank-level values.

Statistical analyses were performed using IBM SPSS Statistics version 27.0 (IBM Corp., Armonk, NY, USA). Differences among the nine treatment combinations were evaluated using one-way analysis of variance followed by Tukey’s honestly significant difference test when the homogeneity-of-variance assumption was satisfied. When Levene’s test indicated heterogeneity of variance, Welch’s analysis of variance followed by the Games–Howell test was used. The main effects of body size, water temperature, and salinity were estimated using a general linear model, with all three factors treated as categorical fixed effects. Because the L_9_ design did not permit independent estimation of interactions, only additive main effects were included, and the results were interpreted as screening estimates. F statistics, *p* values, and partial η^2^ were reported for the main effects, and model-based 95% confidence intervals for the factor-level estimated marginal means are provided in Supplementary Table S1. Residual normality and homogeneity of variance were assessed using the Shapiro–Wilk and Levene’s tests, respectively. Range analysis was retained for descriptive comparison only. Because the analyses were exploratory and conducted within an orthogonal screening framework, no global adjustment was applied across the multiple endpoint-by-factor tests. The reported *p* values should therefore be interpreted as nominal and exploratory rather than confirmatory. Statistical significance was set at *p* < 0.05.

## 3. Results

### 3.1. Hepatic SOD, ALP, and ACP Activities

**Figure 1:**
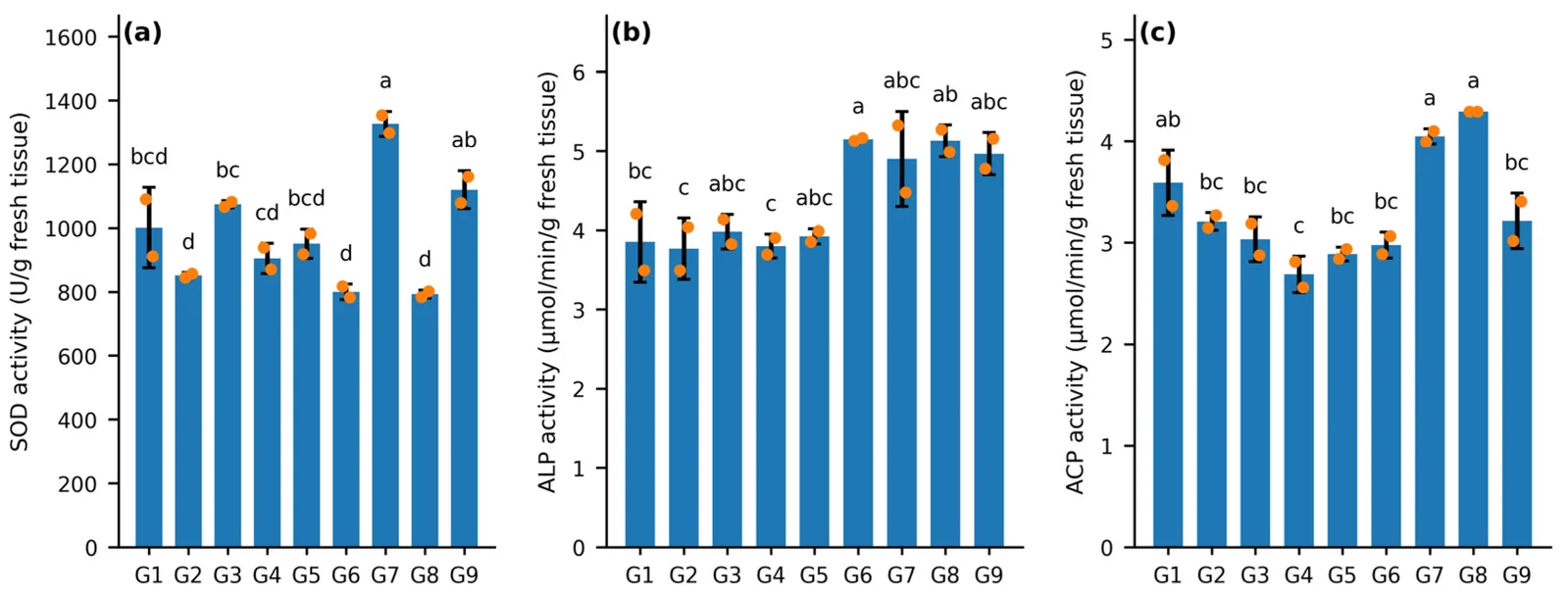
(**a**) Hepatic superoxide dismutase (SOD), (**b**) alkaline phosphatase (ALP), and (**c**) acid phosphatase (ACP) activities in rainbow trout subjected to different treatment combinations. Bars represent the mean ± standard deviation of two tank-level values per treatment. Each point represents one tank-level value calculated as the mean of three fish sampled from the same tank (*n* = 2 tanks per treatment). Different lowercase letters indicate significant differences among treatment combinations based on one-way analysis of variance followed by Tukey’s honestly significant difference test (*p* < 0.05). The experimental conditions corresponding to G1–G9 are provided in Table 1.

The nine treatment combinations differed significantly in hepatic SOD, ALP, and ACP activities (SOD: F(8, 9) = 20.536, *p* < 0.001; ALP: F(8, 9) = 7.361, *p* = 0.004; ACP: F(8, 9) = 18.127, *p* < 0.001; Figure 1). The highest mean SOD activity was observed in G7 (1326.25 U/g), whereas the lowest values occurred in G8 and G6. ALP activity was highest in G6 (5.15 μmol/min/g), closely followed by G8, and was lowest in G2. ACP activity was highest in G8 (4.29 μmol/min/g), followed by G7, and was lowest in G4. The treatment-level patterns were not consistent among the three hepatic enzymes.

### 3.2. Plasma MDA, ALT, and AST

**Figure 2:**
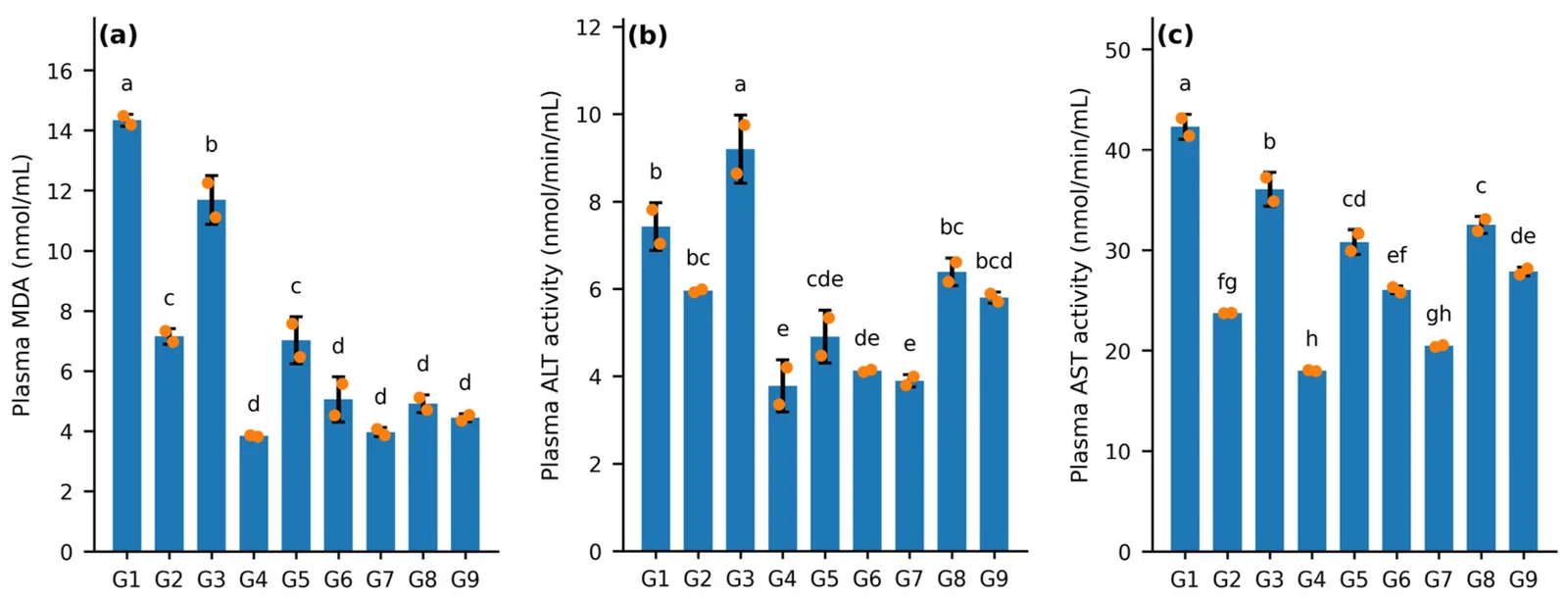
(**a**) Plasma malondialdehyde (MDA) concentration and (**b**) alanine aminotransferase (ALT) and (**c**) aspartate aminotransferase (AST) activities in rainbow trout subjected to different treatment combinations. Bars represent the mean ± standard deviation of two tank-level values per treatment. Each point represents one tank-level value calculated as the mean of three fish sampled from the same tank (*n* = 2 tanks per treatment). Different lowercase letters indicate significant differences among treatment combinations based on one-way analysis of variance followed by Tukey’s honestly significant difference test (*p* < 0.05). The experimental conditions corresponding to G1–G9 are provided in Table 1.

The nine treatment combinations differed significantly in plasma MDA concentration and ALT and AST activities (MDA: F(8, 9) = 118.941, *p* < 0.001; ALT: F(8, 9) = 32.708, *p* < 0.001; AST: F(8, 9) = 152.386, *p* < 0.001; Figure 2). G1 showed the highest mean MDA concentration (14.34 nmol/mL) and AST activity (42.28 nmol/min/mL), whereas G3 showed the highest ALT activity (9.20 nmol/min/mL). G4 showed the lowest mean values for MDA, ALT, and AST. Although MDA and AST exhibited broadly similar treatment-level patterns, the distribution of ALT activity differed among the remaining treatment combinations.

### 3.3. Plasma Urea Nitrogen and Ammonia

**Figure 3:**
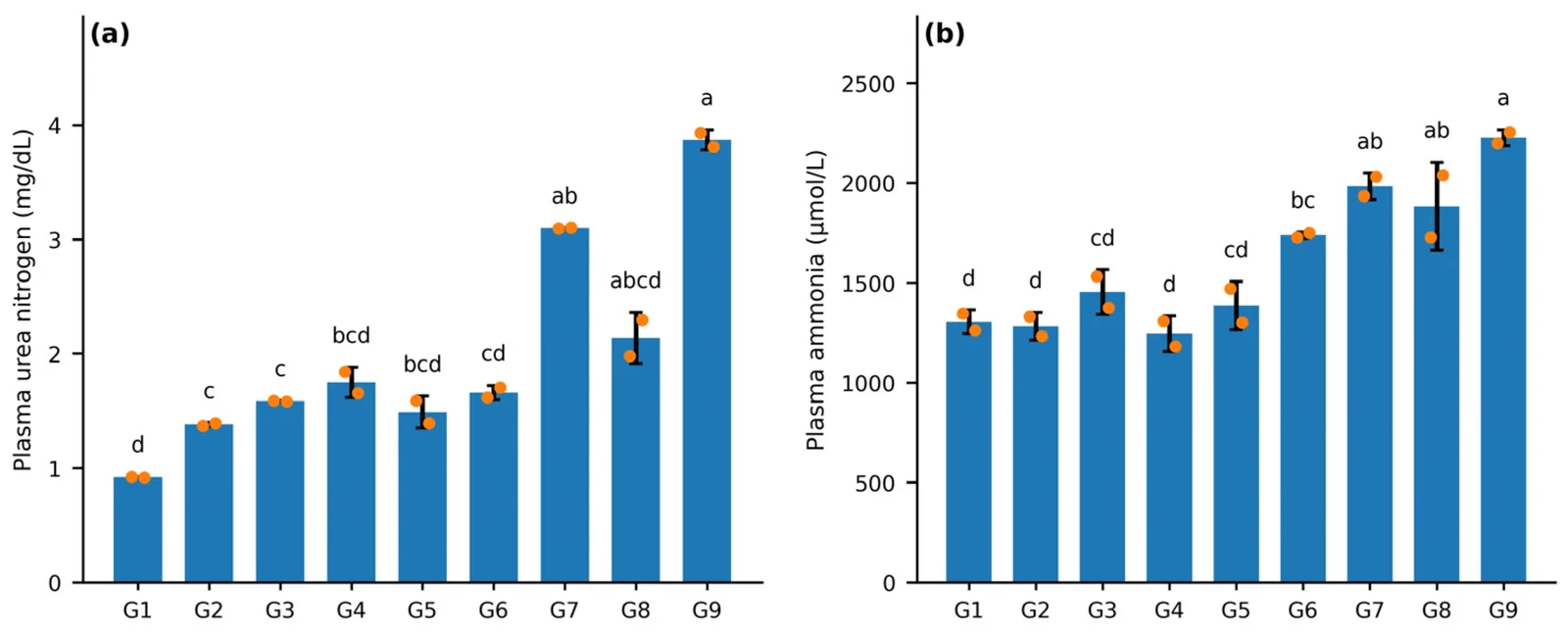
(**a**) Plasma urea nitrogen (BUN) and (**b**) ammonia concentrations in rainbow trout subjected to different treatment combinations. Bars represent the mean ± standard deviation of two tank-level values per treatment. Each point represents one tank-level value calculated as the mean of three fish sampled from the same tank (*n* = 2 tanks per treatment). Different lowercase letters indicate significant differences among treatment combinations. For BUN, comparisons were based on Welch’s analysis of variance followed by the Games–Howell test; for ammonia, comparisons were based on one-way analysis of variance followed by Tukey’s honestly significant difference test (*p* < 0.05). The experimental conditions corresponding to G1–G9 are provided in Table 1.

The nine treatment combinations differed significantly in plasma urea nitrogen and ammonia concentrations (urea nitrogen: Welch’s F(8, 3.610) = 15,342.741, *p* < 0.001; ammonia: F(8, 9) = 23.418, *p* < 0.001; Figure 3). Both variables showed their highest mean values in G9, reaching 3.87 mg/dL for urea nitrogen and 2226.41 μmol/L for ammonia. The lowest urea nitrogen concentration was observed in G1, whereas the lowest ammonia concentration occurred in G4. Mean concentrations of both variables were generally higher in the nominal 500 g groups than in the nominal 100 g groups, although no consistent treatment-level gradient was evident for temperature or salinity.

### 3.4. Orthogonal Range Analysis of Physiological and Biochemical Variables

The descriptive orthogonal range analysis is summarized in Table 2. For the hepatic endpoints, temperature produced the largest R value for SOD, whereas body size produced the largest R values for ALP and ACP. Among the plasma endpoints, body size produced the largest R values for MDA, BUN, ammonia, and ALT. For AST, the R values for temperature, salinity, and body size were relatively similar, although temperature produced the largest value. Overall, the factor-level patterns differed among endpoints and did not indicate a uniform directional response across physiological pathways. Because the R values describe only the spread among factor-level means, they were not treated as evidence of statistical significance and were interpreted together with the tank-level main-effects analysis.

### 3.5. Tank-Level Orthogonal Main-Effects Analysis

The tank-level main-effects analysis is summarized in Table 3. Estimated marginal means and model-based 95% confidence intervals for each factor level are provided in Supplementary Table S1. For the hepatic variables, water temperature was significantly associated with SOD activity, whereas body size was significantly associated with ALP activity. ACP activity was significantly associated with both body size and temperature. No significant salinity effects were detected for SOD, ALP, or ACP.

Among the plasma variables, MDA concentration and AST activity were significantly associated with body size, temperature, and salinity. BUN concentration was associated with body size and temperature, while ammonia concentration was associated only with body size. ALT activity was significantly associated with body size and salinity but not with temperature. The temperature associations identified for ACP and BUN were interpreted cautiously because only two tank-level observations were available per treatment combination and interactions could not be independently estimated.

Overall, body size was associated with the largest number of biochemical endpoints, including ALP, ACP, MDA, BUN, ammonia, ALT, and AST. Temperature and salinity showed more selective associations, particularly for MDA and AST. Because interactions could not be independently estimated in the L_9_ design, these results represent orthogonal screening estimates and should not be interpreted as interaction-adjusted causal effects.

## 4. Discussion

### 4.1. Antioxidant Defense and Lipid Peroxidation

The balance between reactive oxygen species production and antioxidant defense can be altered by changes in environmental conditions. Superoxide dismutase is a major enzymatic component of the antioxidant system, whereas MDA is a product of membrane lipid peroxidation; both are commonly used to assess oxidative status in fish [16,17,18]. However, increased SOD activity primarily reflects modulation of antioxidant defense and does not necessarily indicate tissue injury. Similarly, variation in MDA should be interpreted in conjunction with other physiological variables rather than as an isolated indicator. In the present study, SOD was measured in liver tissue, whereas MDA was measured in plasma; therefore, the two variables represent different sample matrices and different components of oxidative status.

Higher water temperatures generally increase oxygen demand and metabolic activity in rainbow trout, potentially requiring a corresponding adjustment of antioxidant defenses [11,12,16]. Consistent with this descriptive pattern, the tank-level screening analysis identified a significant association between temperature and SOD activity, whereas the associations with body size and salinity were not statistically significant. However, the response was not monotonic across the three temperature levels, and the result should not be interpreted as evidence that a particular tested temperature was uniformly beneficial or detrimental.

The distribution of plasma MDA differed from that of hepatic SOD. Although G7 exhibited the highest SOD activity, its MDA concentration remained comparatively low. Enhanced SOD activity may contribute to the removal of superoxide radicals and thereby limit subsequent lipid peroxidation. Nevertheless, the contrasting pattern between hepatic SOD and plasma MDA does not establish a direct mechanistic relationship between the two variables. MDA accumulation may also be affected by body size, metabolic status, nutritional condition, and tissue-specific processes, which may explain why the two variables did not change in parallel.

Body size produced the largest descriptive R value for MDA, while the tank-level screening analysis identified significant main-effect estimates for body size, temperature, and salinity. The nominal 100 g level showed the highest factor-level mean, whereas lower MDA means were observed at 15 °C and 20‰ than at the other tested levels. Previous studies of salmonids have shown that fish of different sizes may differ in energy reserves, metabolic allocation, and physiological capacity to respond to environmental change [10,13,14,15]. Such size-related differences may influence the production or clearance of lipid peroxidation products. Because interactions could not be independently estimated in the L_9_ design, the observed MDA pattern cannot be attributed exclusively to any single factor. Because catalase, glutathione peroxidase, total antioxidant capacity, and other oxidative biomarkers were not measured, the processes underlying the association between body-size class and MDA could not be determined from the present data. Overall, SOD and MDA represented different aspects of oxidative status, and neither variable alone was sufficient to rank the overall oxidative burden of the nine treatment combinations.

### 4.2. Hepatic Phosphatase Activities

Alkaline phosphatase and ACP are hydrolytic enzymes involved in phosphate metabolism, lysosomal digestion, and nonspecific immune responses in fish [19,20]. ALP participates in phosphate-group hydrolysis and membrane-associated transport processes, whereas ACP is primarily associated with lysosomes and contributes to intracellular digestion and the degradation of foreign or damaged material. Environmental changes may alter the activities of both enzymes, although higher activity should not automatically be interpreted as enhanced immune competence.

The descriptive range ranking was body size > temperature > salinity, and the highest factor-level mean occurred at the nominal 500 g level. Consistent with this pattern, the tank-level screening analysis identified a significant association between body-size class and ALP activity, whereas the associations with temperature and salinity were not statistically significant. Fish of different sizes may differ in hepatic development, basal metabolism, and energy allocation, all of which could contribute to variation in ALP activity [13,14,15,20]. Because ALP is involved in both metabolic and immune-related processes, the relatively high activity recorded at the nominal 500 g level should not be interpreted solely as evidence of stronger innate immunity.

Although the range ranking for ACP was identical to that for ALP, the treatment-level distributions of the two phosphatases were not fully consistent. For example, G6 showed the highest ALP activity but only an intermediate ACP value. This divergence is biologically plausible because ALP and ACP differ in their cellular localization and functional roles. Salinity produced the smallest descriptive R values for both phosphatases, and neither enzyme showed a consistent gradient across the tested range of 10–30‰. The tank-level analysis identified significant associations of body-size class and temperature with ACP activity, whereas the association with salinity was not statistically significant. However, the temperature association for ACP should be interpreted cautiously because only two tank-level observations were available per treatment combination and the L_9_ design could not independently estimate interactions.

Taken together, the revised analysis indicates that body size was associated with both hepatic phosphatase activities, whereas temperature was associated with ACP only. Because interactions could not be independently estimated and only two tank-level observations were available per treatment combination, the ACP temperature association should not be interpreted as a definitive independent response. These phosphatase activities are most appropriately interpreted as supplementary indicators of physiological status and should be considered together with oxidative, transaminase, and nitrogen-metabolism variables [19,20].

### 4.3. Plasma ALT and AST Activities

Alanine aminotransferase and AST participate in amino acid transamination and are closely associated with protein, carbohydrate, and energy metabolism. Their activities in blood may change in response to altered tissue metabolism or cell membrane permeability [21,22]. However, neither enzyme is restricted to the liver, and AST is also abundant in tissues such as skeletal muscle. Consequently, an increase in plasma aminotransferase activity cannot, by itself, be regarded as evidence of hepatic injury.

The descriptive range ranking for ALT was body size > salinity > temperature, and the highest factor-level mean was observed at the nominal 100 g level. Consistent with this pattern, the tank-level screening analysis identified significant associations of body size and salinity with ALT activity, whereas the temperature association was not statistically significant. G3 combined the nominal 100 g body-size level with the highest temperature and salinity levels. Both elevated temperature and increased salinity may increase metabolic and ion-regulatory demands in rainbow trout [3,4,5,6,12]. However, the L_9_ orthogonal array did not allow the independent estimation of interactions among factors. The high ALT activity in G3 therefore cannot be attributed specifically to a synergistic interaction between high temperature and high salinity.

The R values for temperature, salinity, and body size were relatively similar, with temperature showing only a slightly larger value. The tank-level screening analysis identified significant main-effect estimates for body size, temperature, and salinity. Because the three descriptive R values were similar and interactions could not be independently estimated, temperature should not be considered substantially more influential than body size or salinity solely on the basis of the range analysis. Instead, AST appears to have been responsive to multiple intrinsic and environmental factors within the tested screening framework.

MDA, ALT, and AST also differed in their treatment-level patterns. G1 showed the highest MDA and AST values, whereas its ALT activity was lower than that of G3. In contrast, G3 had the highest ALT activity together with a relatively high MDA concentration. Lipid peroxidation, amino acid transamination, and the release or redistribution of enzymes in blood do not necessarily occur concurrently. Plasma aminotransferase activities may also reflect contributions from several tissues and differences in individual metabolic status [21,22]. The broader statistical response of AST compared with ALT may indicate differences in tissue distribution, cellular localization, and sensitivity to the tested treatment conditions. Accordingly, ALT and AST were used here as supplementary indicators of tissue and intermediary metabolism rather than as direct diagnostic markers of liver damage.

### 4.4. Plasma Urea Nitrogen and Ammonia

Ammonia is the principal nitrogenous waste product in most teleost fish. It is produced mainly through the catabolism of proteins and amino acids, transported through the circulation, and excreted predominantly across the gills. Plasma ammonia concentration therefore reflects the balance among internal ammonia production, tissue transport, and branchial excretion, each of which may be affected by feeding status, temperature, salinity, and acid–base regulation. Rainbow trout also possess functional pathways for urea synthesis and excretion; consequently, BUN can provide complementary information on nitrogen metabolism [23,24,25,26]. The physiological interpretation of BUN in fish differs from its conventional clinical use in mammals and should not be restricted to the assessment of renal function.

Body size produced the largest R value for BUN, and the nominal 500 g level showed a higher level mean than the nominal 100 and 250 g levels. Larger fish may differ from smaller individuals in tissue mass, protein turnover, energy reserves, and nitrogen metabolism, potentially resulting in different rates of urea production, transport, or excretion. Consistent with the descriptive range pattern, the tank-level screening analysis identified significant associations of body size and temperature with BUN, whereas the salinity association was not statistically significant. However, the temperature association should be interpreted cautiously because only two tank-level observations were available per treatment combination and interactions could not be independently estimated.

The treatment distribution of plasma ammonia broadly resembled that of BUN. Body size again showed the largest R value, followed by salinity, whereas temperature produced only a small range. Notably, G9 combined 15 °C with 10‰ salinity rather than the highest temperature or salinity level. Thus, the elevated ammonia concentration in this group cannot be attributed simply to increasing temperature or salinity. The tank-level screening analysis identified a significant association between body-size class and plasma ammonia, whereas the associations with temperature and salinity were not statistically significant.

The concurrent elevation of BUN and ammonia at the nominal 500 g level indicates a distinct circulating nitrogen-metabolism profile among the body-size classes; however, the underlying processes could not be determined from the measured endpoints. Consistent with this pattern, body size was significantly associated with both BUN and ammonia. Temperature was associated with BUN but not with ammonia; however, the BUN temperature association should be interpreted cautiously because of the limited tank replication and the inability to estimate interactions. In the absence of measurements of branchial ammonia excretion, blood acid–base status, or ammonia-transport proteins, it is not possible to determine whether the higher plasma ammonia concentrations resulted from increased production, altered transport, or reduced excretion.

Because both variables were measured in plasma, they represent circulating nitrogen-metabolism status rather than direct rates of nitrogen production or excretion. In this study, plasma urea nitrogen and ammonia were used primarily for relative comparisons among treatment combinations and to provide complementary information on nitrogen metabolism alongside plasma aminotransferase activities [23,24,25,26].

### 4.5. Integrated Responses, Scope, and Limitations

The range rankings differed among the eight physiological and biochemical variables. Temperature produced the largest descriptive R values for SOD and AST, although the R values of temperature, salinity, and body size for AST were relatively similar. Body size produced the largest R values for ALP, ACP, MDA, BUN, ammonia, and ALT, whereas salinity generally showed smaller ranges. These differences indicate that biomarkers associated with antioxidant defense, phosphatase activity, transamination, and nitrogen metabolism did not respond uniformly to the three experimental factors. The tank-level analysis further indicated that body size was associated with a broader range of endpoints, whereas temperature and salinity showed more selective associations.

Body-size-associated patterns were particularly evident. The nominal 500 g level showed higher level means for ALP, ACP, BUN, and ammonia, whereas the nominal 100 g level showed higher level means for MDA, ALT, and AST. Previous studies have demonstrated that body size is associated with energy reserves, organ development, metabolic allocation, and seawater adaptability in salmonids [13,14,15,20]. However, the present results did not show a uniform advantage or disadvantage for any body-size class. Larger fish did not exhibit lower values for all measured biochemical variables, nor did smaller fish consistently exhibit higher values across all endpoints. Moreover, the three nominal body-size levels may also have differed in age, developmental stage, physiological condition, and energy reserves. Although the initial biomass per tank was broadly comparable among the nominal body-size levels, the numbers of fish stocked per tank differed substantially, with 140, 40, and 14 fish stocked for the nominal 100, 250, and 500 g levels, respectively. Thus, fish-number density and the associated social, feeding, and waste-production conditions may also have contributed to the observed biochemical variation. The identified body-size associations therefore cannot be attributed exclusively to body mass. Instead, the body-size classes appeared to differ in the physiological processes represented by the measured biomarkers rather than in a single overall direction of response.

Temperature produced the largest descriptive R values for SOD and AST, consistent with previous evidence that antioxidant and transamination-related processes in rainbow trout are sensitive to thermal conditions [11,12,16,22]. The tank-level analysis identified significant temperature associations for SOD, ACP, MDA, BUN, and AST. Among these, the ACP and BUN associations were treated as secondary findings because of the limited tank replication and the inability to estimate interactions. Similarly, salinity did not produce a consistent biomarker gradient. The 30‰ treatments did not show the highest values for all variables, and several maxima occurred at 10 or 20‰. Previous studies have likewise shown that physiological responses to salinity depend on acclimation conditions and the specific endpoint examined [3,4,5,6]. Salinity was significantly associated with MDA, ALT, and AST but not with the remaining endpoints, further supporting a pathway-specific rather than uniform biochemical response.

The revised tank-level analysis identified significant body-size associations for ALP, ACP, MDA, BUN, ammonia, ALT, and AST. Temperature and salinity showed fewer but statistically supported associations, particularly for MDA and AST. These results strengthen the evidence that the observed biochemical variation was not restricted to a single oxidative marker. Nevertheless, the range analysis remained descriptive, and the main effects were interpreted as orthogonal screening estimates because interactions could not be independently estimated. The main biological contribution of the study is therefore not the identification of a universally optimal treatment, but evidence that the nominal body-size classes were associated with distinct cross-pathway biochemical profiles across the tested temperature–salinity combinations.

The experiment was conducted in flow-through systems under generally stable water-quality conditions. All biochemical measurements were obtained at the end of the 60-day trial and therefore represented endpoint biochemical status. They did not capture responses occurring during the initial or intermediate stages of salinity acclimation. Moreover, no 0‰ freshwater control was included. The results are therefore restricted to comparisons among the nine combinations of 10–30‰ salinity and cannot be used to determine whether individual biomarkers increased or decreased relative to freshwater baseline conditions. Mortality occurred during the experiment, and biochemical samples were collected from fish surviving to the scheduled endpoint. Consequently, the measured profiles may partly reflect survivor selection, particularly in treatment combinations with relatively high mortality, and should not be used alone to infer treatment safety or overall culture suitability.

Although the tank was treated as the experimental unit, only two independent tank-level observations were available for each treatment combination, which limited statistical power and the precision of the estimated main-effect associations. The L_9_ orthogonal design was intended to compare the relative main effects of body size, temperature, and salinity and could not independently estimate their interactions. Because plasma osmolality, major plasma ions, branchial Na^+^/K^+^-ATPase activity, cortisol, hematological variables, and gill histology were not measured, the observed biochemical patterns cannot be linked directly to osmoregulatory performance or tissue-level mechanisms. Because actual feed intake was not quantified and growth performance was not included in the present analysis, their possible contribution to the observed biochemical differences could not be evaluated. In addition, because no global multiplicity adjustment was applied across the endpoint-by-factor tests, the reported associations should be regarded as exploratory findings requiring confirmation in independently replicated experiments. Future studies should use a full-factorial design focused on the factor levels identified as potentially influential in the present experiment. Repeated sampling during the acclimation period, together with measurements of blood ions, plasma osmolality, branchial Na^+^/K^+^-ATPase activity, cortisol, hematological variables, ammonia-excretion rates, and tissue histology, would provide a more mechanistic understanding of the physiological responses associated with body-size class, temperature, and salinity exposure in rainbow trout.

## 5. Conclusions

The endpoint biochemical responses and associated physiological status of rainbow trout varied among the nine combinations of body size, water temperature, and salinity evaluated using the L_9_ orthogonal array. The different treatment-level patterns indicated that antioxidant defense, phosphatase activity, transamination, and nitrogen metabolism did not respond in a coordinated manner to the experimental conditions. The tank-level screening analysis indicated that body size was associated with the broadest range of biochemical endpoints, including hepatic ALP and ACP activities and plasma MDA, urea nitrogen, ammonia, ALT, and AST. Temperature and salinity showed more selective associations: temperature was associated with hepatic SOD and ACP and plasma MDA, BUN, and AST, whereas salinity was associated with plasma MDA, ALT, and AST.

These findings suggest that the body-size classes differed across several physiological pathways rather than in a single overall direction of response. However, because interactions could not be independently estimated in the L_9_ design, the identified main effects should be interpreted as screening associations rather than as isolated causal effects. Because no freshwater control was included and all biochemical variables were measured only at the end of the 60-day trial, the present findings are restricted to comparisons among the tested combinations of body-size class, temperature, and salinity within the range of 10–30‰ and describe endpoint biochemical status rather than direct osmoregulatory performance or the temporal course of acclimation. Further replicated full-factorial studies incorporating freshwater controls, repeated sampling, plasma osmolality, blood-ion concentrations, branchial Na^+^/K^+^-ATPase activity, ammonia-excretion rates, and tissue histology are required to clarify the independent and interactive effects of body size, temperature, and salinity on physiological responses to saline environments. Overall, these findings highlight the importance of incorporating body-size class into experimental designs and biochemical assessments of rainbow trout under brackish and saline culture conditions.

## Figures and Tables

**Table 1:** L_9_ (3^4^) orthogonal array used for the rainbow trout salinity-acclimation trial.

Group	Body Size (g)	Temperature (°C)	Salinity (‰)
G1	100	10	10
G2	100	15	20
G3	100	20	30
G4	250	15	30
G5	250	20	10
G6	250	10	20
G7	500	20	20
G8	500	10	30
G9	500	15	10

Note: The values of 100, 250, and 500 g represent the predefined body-size factor levels used in the orthogonal experimental design. The actual initial mean body weights of fish assigned to these levels are reported in Section 2.1.

**Table 2:** Descriptive orthogonal range analysis of body size, water temperature, and salinity for hepatic and plasma biochemical endpoints in rainbow trout.

Variable	Factor	*k*_1_	*k*_2_	*k*_3_	*R*
SOD (U/g)	Body size	975.68	885.14	1079.69	194.55
	Temperature	864.63	958.75	1117.13	252.50
	Salinity	1023.93	992.58	924.00	99.93
ALP (μmol/min/g)	Body size	3.866	4.289	4.998	1.132
	Temperature	4.709	4.177	4.267	0.532
	Salinity	4.246	4.604	4.302	0.358
ACP (μmol/min/g)	Body size	3.277	2.851	3.851	1.001
	Temperature	3.620	3.036	3.323	0.584
	Salinity	3.231	3.411	3.338	0.180
MDA (nmol/mL)	Body size	11.058	5.304	4.441	6.617
	Temperature	8.101	5.146	7.557	2.955
	Salinity	8.601	5.389	6.814	3.212
BUN (mg/dL)	Body size	1.295	1.633	3.036	1.741
	Temperature	1.573	2.334	2.058	0.761
	Salinity	2.094	2.047	1.824	0.270
Ammonia (μmol/L)	Body size	1346.26	1456.36	2030.76	684.49
	Temperature	1641.81	1584.29	1607.29	57.52
	Salinity	1638.77	1667.49	1527.13	140.36
ALT (nmol/min/mL)	Body size	7.528	4.271	5.362	3.257
	Temperature	5.980	5.179	6.001	0.822
	Salinity	6.043	4.660	6.457	1.797
AST (nmol/min/mL)	Body size	34.022	24.948	26.952	9.074
	Temperature	33.611	23.207	29.104	10.403
	Salinity	33.649	23.416	28.857	10.234

Notes: *k*_1_, *k*_2_, and *k*_3_ represent the mean responses at the first, second, and third levels of each factor, respectively. Each treatment mean was calculated from two tank-level values. For body size, levels 1, 2, and 3 corresponded to the nominal 100, 250, and 500 g levels, respectively. For temperature, levels 1, 2, and 3 corresponded to 10, 15, and 20 °C, respectively. For salinity, levels 1, 2, and 3 corresponded to 10, 20, and 30‰, respectively. *R* was calculated as the difference between the maximum and minimum level means. *R* values were used descriptively and should only be compared among factors within the same endpoint.

**Table 3:** Tank-level orthogonal main-effects analysis of body size, water temperature, and salinity for physiological and biochemical variables in rainbow trout.

Variable	Factor	F (2, 11)	*p*	Partial η^2^
SOD	Body size	3.800	0.056	0.409
	Temperature	6.529	**0.014**	0.543
	Salinity	1.047	0.383	0.160
ALP	Body size	11.602	**0.002**	0.678
	Temperature	2.877	0.099	0.343
	Salinity	1.318	0.307	0.193
ACP	Body size	19.230	**<0.001**	0.778
	Temperature	6.502	**0.014**	0.542
	Salinity	0.625	0.553	0.102
MDA	Body size	159.299	**<0.001**	0.967
	Temperature	30.461	**<0.001**	0.847
	Salinity	31.885	**<0.001**	0.853
BUN	Body size	34.692	**<0.001**	0.863
	Temperature	6.043	**0.017**	0.524
	Salinity	0.845	0.456	0.133
Ammonia	Body size	20.274	**<0.001**	0.787
	Temperature	0.126	0.883	0.022
	Salinity	0.825	0.464	0.130
ALT	Body size	25.278	**<0.001**	0.821
	Temperature	2.021	0.179	0.269
	Salinity	8.143	**0.007**	0.597
AST	Body size	41.047	**<0.001**	0.882
	Temperature	49.170	**<0.001**	0.899
	Salinity	47.364	**<0.001**	0.896

Notes: The analysis was based on 18 tank-level observations. For each treatment combination, the three fish sampled from the same tank were averaged to obtain one tank-level value, resulting in two tank-level observations per treatment. Body size, water temperature, and salinity were included as categorical fixed effects in an additive general linear model. Each factor had two degrees of freedom, and the residual error had 11 degrees of freedom. Partial η^2^ represents the effect size. Interaction terms were not included because they could not be independently estimated using the L_9_ design. Bold values indicate statistical significance at *p* < 0.05. The temperature associations for ACP and BUN should be interpreted cautiously because only two tank-level observations were available per treatment combination and interactions could not be independently estimated in the L_9_ design. Partial η^2^ values are reported as descriptive effect-size estimates and should not be interpreted as precise measures of biological effect magnitude because of the limited tank-level replication.

## Data Availability

The raw data supporting the conclusions of this article will be made available by the authors on reasonable request.

## Supplementary Materials

The following supporting information can be downloaded at: https://www.mdpi.com/article/10.3390/biology15161383/s1, Table S1. Estimated marginal means and model-based 95% confidence intervals for each level of body size, water temperature, and salinity. Table S2. Mortality percentages of rainbow trout across the nine treatment combinations. Table S3. Actual mean water temperature and salinity for the nine treatment combinations.
